# Supplementary material for: Molecular polariton electroabsorption
Source: Nat Commun. 2022 Dec 24;13:7937. doi: 10.1038/s41467-022-35589-4 (PMC9789964; doi:10.1038/s41467-022-35589-4)
Supplement: Supplementary file 2 — Supplementary Information [file 41467_2022_35589_MOESM2_ESM.pdf]

Supplementary Information:

Molecular polariton electroabsorption

Chiao-Yu Cheng, Nina Krainova, Alyssa Brigeman, Ajay Khanna, Sapana Shedge, Christine Isborn, Joel Yuen-Zhou, and Noel C. Giebink

S1. Current-voltage relationships

A typical current-voltage characteristic recorded for a 40 wt% SQ:NPB microcavity device is shown in Fig. S1. The device conducts very little current and essentially behaves as a capacitor; the apparent open-circuit voltage results from chemical capacitance (i.e. charging of traps) and changes depending on the direction of the bias sweep.

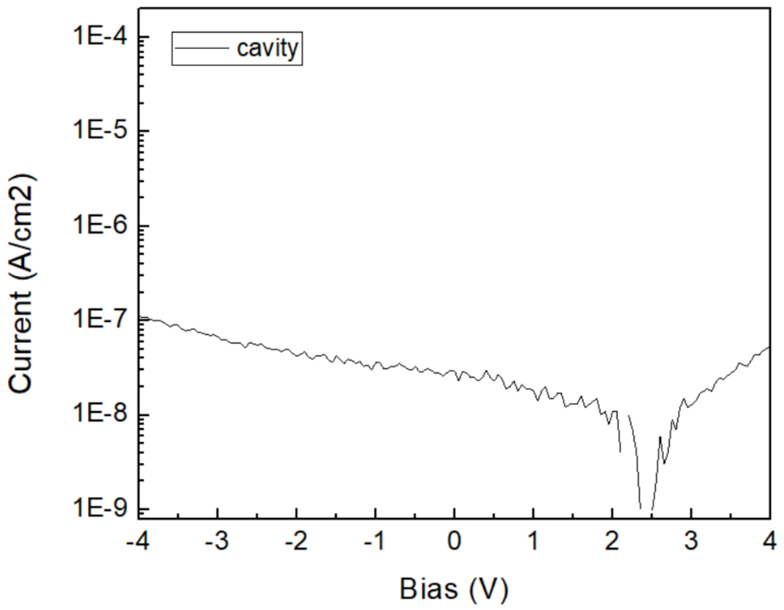

Figure S1. Current-voltage characteristic for the negatively-detuned 40 wt% SQ microcavity.

S2. Transfer matrix method for simulating EA spectra

The complex refractive index dispersion (n) of the SQ films is described as a function of energy (E) using a generic Lorentz oscillator model:

$$n^2 = \varepsilon_{\rm r} = \varepsilon_\infty + \sum_j \frac{f_j}{E_j^2 - E^2 - i\Gamma_j E}, \tag{S1}$$

where $\varepsilon_{\rm r} = 1 + \chi$ is the linear dielectric function, ε_∞ is the high frequency dielectric constant, and E_j, f_j, and Γ_j are the energy, amplitude, and broadening of the j-th oscillator, respectively. In the presence of an applied electric field, F, the refractive index may be modified, most generally through a change in oscillator energy ($E_j \to E_j + \Delta E_j$), amplitude ($f_j \to f_j + \Delta f_j$), or broadening ($\Gamma_j \to \Gamma_j + \Delta\Gamma_j$). From the perspective of nonlinear optics, the change in susceptibility that results, $\Delta\chi$, is related to the third order nonlinear susceptibility via $\chi^{(3)}(\omega, 0,0) = \Delta\chi/(3F^2)$, where $\omega = E/\hbar$ is the angular frequency[1].

Using the transfer matrix model to calculate the sample reflectivity with and without the field-modified refractive index, it is then straightforward to obtain the differential reflectivity, $\Delta R/R \equiv [R(F) - R(0)]/R(0)$ that is measured in experiment. Other field-induced changes such as electrostriction (i.e. a field-induced decrease in cavity length) are also straightforward to account for, though we found no evidence that this is significant in our data analysis. Additionally, the applied field could produce uniaxial anisotropy in the refractive index, but this would not impact the s-polarized reflectivity that we study here.

S3. Polariton dispersion of 5 wt% SQ:NPB cavities

Figure S2 shows the polariton dispersion relations obtained from the reflectivity data of the negatively- and positively-detuned 5 wt% SQ:NPB cavities in Fig. 2d,g, respectively. The data are fit using Eqn. (1) considering a single exciton state, which reduces to the standard 2x2 coupled oscillator Hamiltonian:

$$H_{SC}(k) = \begin{bmatrix} E_1 & V_1 \\ V_1 & E_{\mathrm{ph,k}} \end{bmatrix}, \quad \text{(S2)}$$

where the bare cavity mode dispersion $E_{\mathrm{ph,k}} = E_{\mathrm{ph0}}/\sqrt{1-(\sin(\theta)/n_{\mathrm{cav}})^2}$, is parameterized in terms of an effective index, n_{cav} (which includes the mirror reflection phase), and the cavity mode cutoff energy, E_{ph0}. Diagonalizing Eqn. (S2) to obtain the polariton energies, we obtain good agreement for both cavities using $V_1 = 0.1$ eV as shown in Fig. S2.

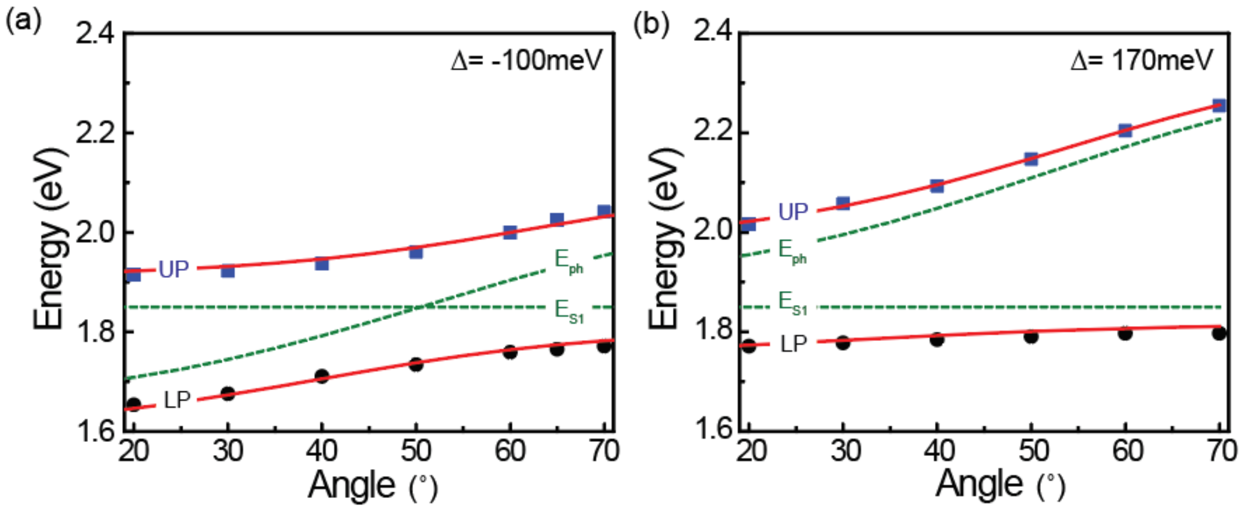

Figure S2. Polariton dispersion relations for the **(a)** negatively- and **(b)** positively-detuned 5 wt% SQ:NPB microcavities. The solid markers are determined from the experimental reflectivity data in Fig. 2d,g and the red solid lines are calculated using Eqn. (S2) with $n_{\mathrm{cav}} = 1.75$ and

$E_{\mathrm{ph0}} = 1.75$ eV (2.02 eV) for the negatively (positively)-detuned cavity. The green dashed lines denote the bare exciton energy and cavity mode dispersion.

S4. Derivative analysis of polariton EA

If the applied field causes a given polariton mode to shift in energy by amount, ΔE, then the field-perturbed reflectivity can be approximated via a Taylor expansion, $R(E - \Delta E) \approx R(E) - (\Delta E)\, dR/dE$, in terms of the original zero-field reflectivity, $R(E)$. Rewriting this expression in terms of the reflectivity difference, $\Delta R = R(E - \Delta E) - R(E)$, and dividing by $R(E)$ then leads to the expression, $\Delta R/R \approx -\Delta E[(1/R)\, dR/dE]$, referred to in the main text, where the proportionality constant to be determined between the experimental electroreflectance ($\Delta R/R$) and the normalized reflectivity derivative $[(1/R)\, dR/dE]$ is $-\Delta E$. This relationship is illustrated in Fig. S3 below.

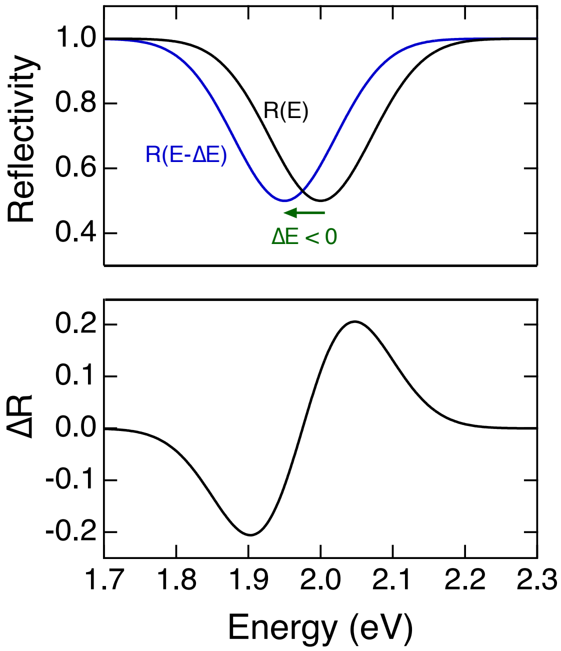

Figure S3. Schematic illustration of the reflectivity difference that results from red-shifting (i.e. $\Delta E < 0$) a typical polariton reflectivity dip.

S5. Electronic structure calculations for squaraine

The impact of an applied field on the SQ exciton transition is examined by computing 'field-off' and 'field-on' linear response time-dependent density functional theory (TDDFT) excitation energies and transition dipole moments within the Tamm-Dancoff approximation. Geometry optimizations and normal mode analysis, dipole moment computation, as well as molecular orbital visualization and TDDFT calculations, are performed in vacuum using the Gaussian electronic structure package version 16 at the B3LYP/6-31G* level of theory. The $S_0 \rightarrow S_1$ bright exciton transition is primarily HOMO to LUMO in character, with the molecular orbitals shown in Fig. S4. Note that the excitation energy is overestimated with TDDFT, partially due to the use of a relatively small basis set. With the molecular axis along the x-direction, the x-component of the transition dipole is the largest, and a field applied in this direction couples to the exciton transition. An applied field along the x-direction of +/-0.01 atomic units (a.u., equivalent to ~51 MV cm^{-1}) shifts the energy of the transition by 0.03-0.04 eV; see Table S1. Fields applied in the y- or z-direction cause minimal change to the transition energy.

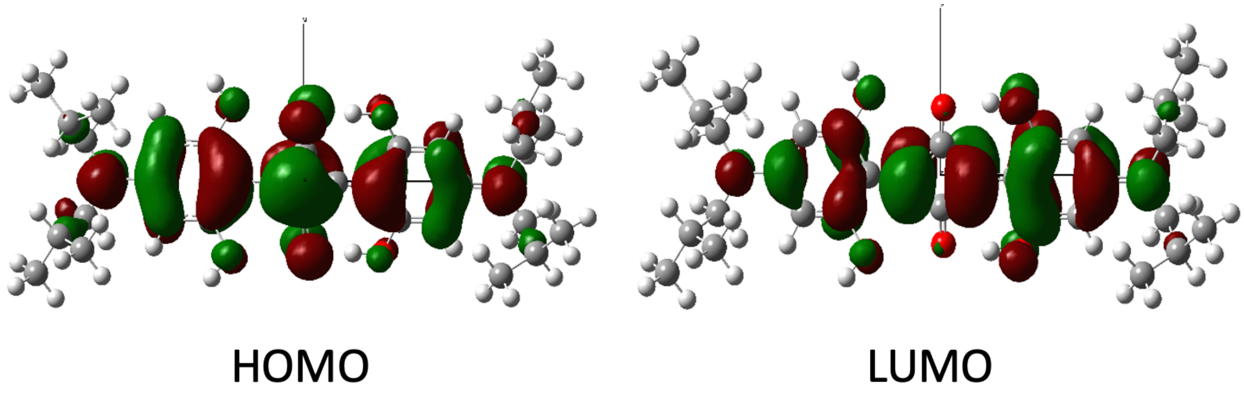

Figure S4. B3LYP/6-31G* molecular orbitals for computed for squaraine in vacuum. The S_0 to S_1 exciton transition is primarily HOMO to LUMO in character.

Table S1. Field-off and field-on TD-B3LYP/6-31G* S_1 excitation energies (eV), transition dipole moments (atomic units), and oscillator strengths for squaraine in vacuum

	Field (a.u.)	ΔE (eV)	μ_x (a.u)	μ_y (a.u)	μ_z (a.u)	f
S_1	0.0	2.41	4.5754	-0.0584	0.1208	1.2380
S_1	X=+0.01	2.38	4.6350	-0.0620	0.1182	1.2513
S_1	X=-0.01	2.45	4.5271	-0.0436	0.1289	1.2304

S6. Equivalence of Hamiltonian and transfer matrix descriptions of polariton EA

As discussed in the main text, the transfer matrix description of polariton EA operates sequentially (first perturbing the exciton transitions and then solving Maxwell's equations to obtain the perturbed polariton modes), whereas the Hamiltonian approach using Eqn. (1) operates with the static electric field and cavity coupling all at once. Though intuitively expected, it is not immediately obvious that these two different approaches yield equivalent results.

To show this, we begin by emphasizing the equivalence of the field-perturbed polariton modes computed in each basis. That is, the polariton energies obtained by diagonalizing Eqn. (1) in the light-matter basis (call them λ_{LM1}, λ_{LM2}, and λ_{LM3}) are equivalent to what one obtains by first diagonalizing the matter Hamiltonian:

$$H_{\mathrm{Matter}} = \begin{bmatrix} E_1 & \mu_{12}F \\ \mu_{12}F & E_2 \end{bmatrix}, \tag{S3}$$

to determine the field-perturbed exciton eigenenergies, $\lambda_{1,2}$, and then coupling these exciton states to the cavity photon via:

$$H'_{SC}(k) = \begin{bmatrix} \lambda_1 & 0 & V'_1 \\ 0 & \lambda_2 & V'_2 \\ V'_1 & V'_2 & E_{\text{ph,k}} \end{bmatrix}, \tag{S4}$$

where V'_1 and V'_2 are the light-matter couplings projected in the field-perturbed matter basis (i.e. $V'_1 = [V_1\ V_2]\mathbf{v}_1$ and $V'_2 = [V_1\ V_2]\mathbf{v}_2$ based on the column eigenvectors, $\mathbf{v}_{1,2}$ of Eqn. (S3)). It is subsequently straightforward to verify that the eigenenergies (as well as the eigenvectors upon corresponding change of basis) of Eqn. (S4) are equivalent to those of Eqn. (1) in the main text.

To see whether this equivalence is preserved when the light-matter coupling in Eqn. (S4) is implemented via the transfer matrix, we carry out a numerical experiment where we assume an idealized system with two exciton transitions (at energies $E_1 = 2.1$ eV and $E_2 = 2.3$ eV) represented by two Lorentz oscillators of equal strength as shown in the optical constant dispersion of Fig S5a. We then implement a 130 nm-thick layer of this material between a 100 nm-thick bottom Ag mirror and a 30 nm-thick semitransparent top Ag mirror and then calculate the resulting angle-dependent reflectivity spectra (Fig. S5b), which exhibit clear lower, middle, and upper polariton branches. Treating this as mock experimental data, we then fit the resulting dispersion of each feature in Fig. S5c with Eqn. (1) at zero field to determine the light-matter interaction energies, $V_1 = V_2 = 0.11$ eV.

To calculate the electroreflectance spectra from the transfer matrix perspective, we determine the effect of the applied field on the two Lorentz oscillators by diagonalizing Eqn. (S3) with a field perturbation $\mu_{12}F = 1$ meV to determine their new energies ($\lambda_{1,2}$) and amplitudes (i.e. $f'_{1,2} = f_{1,2}\left[V'_{1,2}/V_{1,2}\right]^2$ since oscillator strength is proportional to the square of the light-matter interaction energy). Re-simulating the reflectivity with these new oscillator parameters then enables calculation of the differential reflectance spectra (relative to the zero-

field spectra in Fig. S5b) shown in Fig. S5e. To calculate the electroreflectance from the perspective of Eqn. (1), we diagonalize it with and without the field perturbation to determine the associated polariton energy shifts plotted in Fig. S5d. We then fit the original zero-field reflectance in Fig. S5b with three Lorentzian peaks that capture the contribution of each polariton, apply their respective field-induced energy shifts from Fig. S5d, and then calculate the resulting differential reflectivity spectra, which exhibit good agreement with the transfer matrix results in Fig. 5e (blue dotted and black solid lines, respectively). Thus, we conclude that the two approaches to describe polariton EA are equivalent within the error of the multipeak fitting.

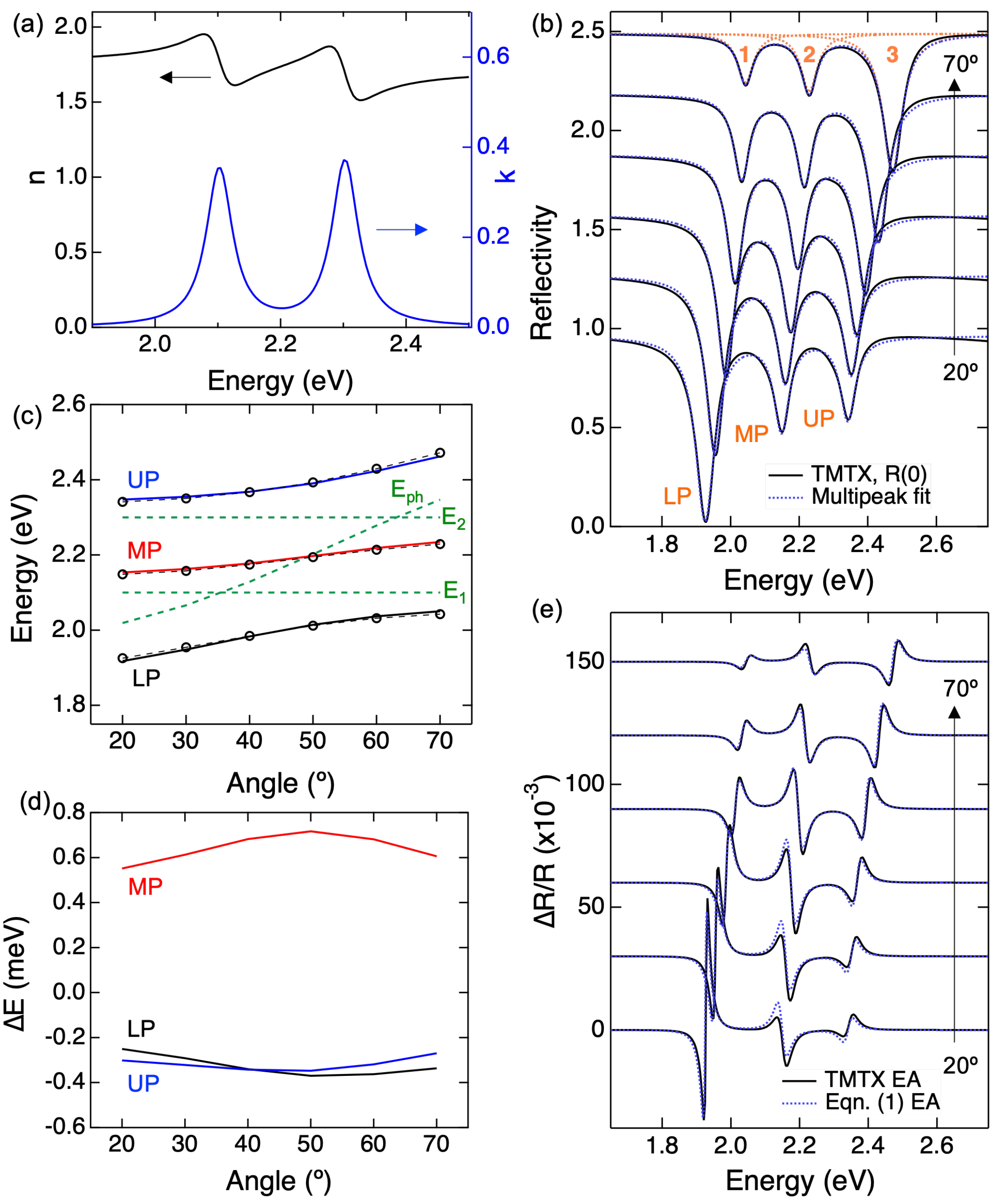

Figure S5. (a) Optical constant dispersion of an idealized organic material with two, equal strength exciton transitions described by Lorentz oscillators. **(b)** Transfer matrix-simulated s-polarized reflectivity spectra for a Ag (30 nm)/organic (130 nm)/Ag (100 nm) microcavity (light is incident on the thin Ag mirror) at different incidence angles. The dotted blue lines are the result of multipeak fitting with three Lorentzian dips (highlighted by the orange dotted lines on

the 70º spectrum) numbered 1, 2, 3 to respectively capture the LP, MP, and UP reflectivity contributions. **(c)** Polariton dispersion determined from the reflectivity minima in (b) (black open circles), together with that calculated from Eqn. (1) at zero field (solid lines) using light-matter coupling strengths $V_1 = V_2 = 0.11$ eV. **(d)** Field-induced polariton energy shifts computed from Eqn. (1) in the presence and absence of the field perturbation. **(e)** Electroreflectance spectra computed via the transfer matrix method (black solid lines) and the Hamiltonian approach (blue dotted lines), where the LP, MP, and UP component peaks from (b) are shifted in energy by the corresponding amounts in (d).

We perform a similar numerical experiment to check the self-consistency of the reflectivity derivative approach used to determine the polariton energy shifts in Fig. 5 of the main text. For this example, we model the same scenario as in Fig. 5c,d, which involves field-induced mixing between the exciton at $E_1 = 1.85$ eV and an upper excited state at $E_2 = 3$ eV. Figure S6a shows the optical constant dispersion in the vicinity of the exciton transition and Fig. S6b shows the angle-dependent reflectivity spectra that are simulated for a microcavity with 160 nm of this organic material placed between a 100 nm-thick bottom Ag mirror and a 30 nm-thick semitransparent top Ag mirror. Figure S6c shows the transfer matrix-simulated differential reflectance spectra following the procedure described above (i.e. based on the oscillator energy shift and amplitude change calculated from Eqn. (S3)), which are similar in lineshape but different in amplitude from the zero-field reflectivity derivative spectra shown in Fig. S6d. After aligning the Hamiltonian-simulated polariton dispersion with that obtained from the transfer matrix reflectivity data (Fig. S6e), we calculate the field-induced polariton shifts from Eqn. (1) and compare them with those extracted by scaling the peaks of the LP and UP reflectivity

derivatives in Fig. S6d to match the mock EA data in Fig. S6c. The agreement between the two calculations shows that the reflectivity derivative method of inferring polariton energy shifts from EA spectra is self-consistent with the shifts obtained directly from the system Hamiltonian.

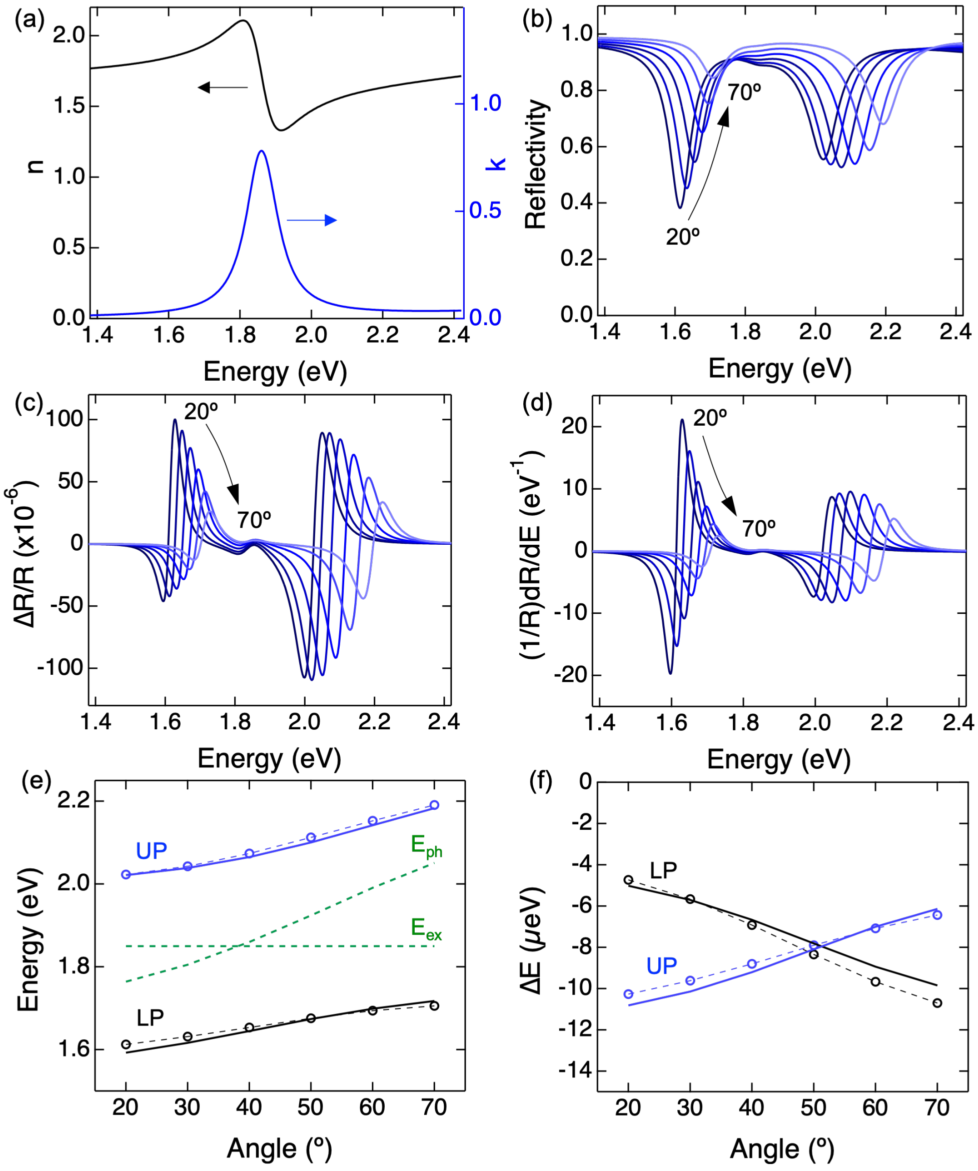

Figure S6. (a) Optical constant dispersion of an idealized organic material with an exciton transition described by a Lorentz oscillator located at 1.85 eV. **(b)** Transfer matrix-simulated s-polarized reflectivity spectra for a Ag (30 nm)/organic (160 nm)/Ag (100 nm) microcavity (light

is incident on the thin Ag mirror) at different incidence angles. **(c)** Electroreflectance spectra computed via the transfer matrix method by adjusting the Lorentz oscillator energy and amplitude based on Eqn. (S3) as described in the text. **(d)** Reflectivity derivative spectra computed from the data in (b). **(e)** Polariton dispersion determined from the reflectivity minima in (b) (black open circles), together with that calculated from Eqn. (1) at zero field (solid lines) using a light-matter coupling strength $V_1 = 0.21$ eV. **(f)** Field-induced polariton energy shifts computed directly from the Hamiltonian (solid lines) compared with those obtained by scaling the LP and UP reflectivity derivatives to match to match the amplitude of their respective features in (c). All of the simulations assume $\mu_{12} = 20$ D and an applied field strength of $F = 10^5$ V cm^{-1}.

S7. Evidence for H-aggregation in SQ:NPB blends

Figure S7a shows the normalized absorption spectra for a series of 100 nm-thick SQ:NPB films, highlighting an increase in the 0-1 high energy vibronic shoulder relative to the 0-0 transition with increasing SQ concentration . This is accompanied by a relative increase in the 0-1 vibronic photoluminescence intensity (Fig. S7b) as well as a rapid decrease in photoluminescence quantum yield (Fig. S7c). All of these observations are consistent with the signatures of H-aggregate formation described in Ref. [2] and have also been observed for SQ in other host matrices[3]. We note that the broadening on the low energy side of the main exciton transition in Fig. S7a can be reproduced by including a weak absorption band located at 1.76 eV that would be consistent with the (nearly dark) lower H-aggregate inferred from the polariton EA analysis, though it is not possible to reliably constrain this peak in a fit.

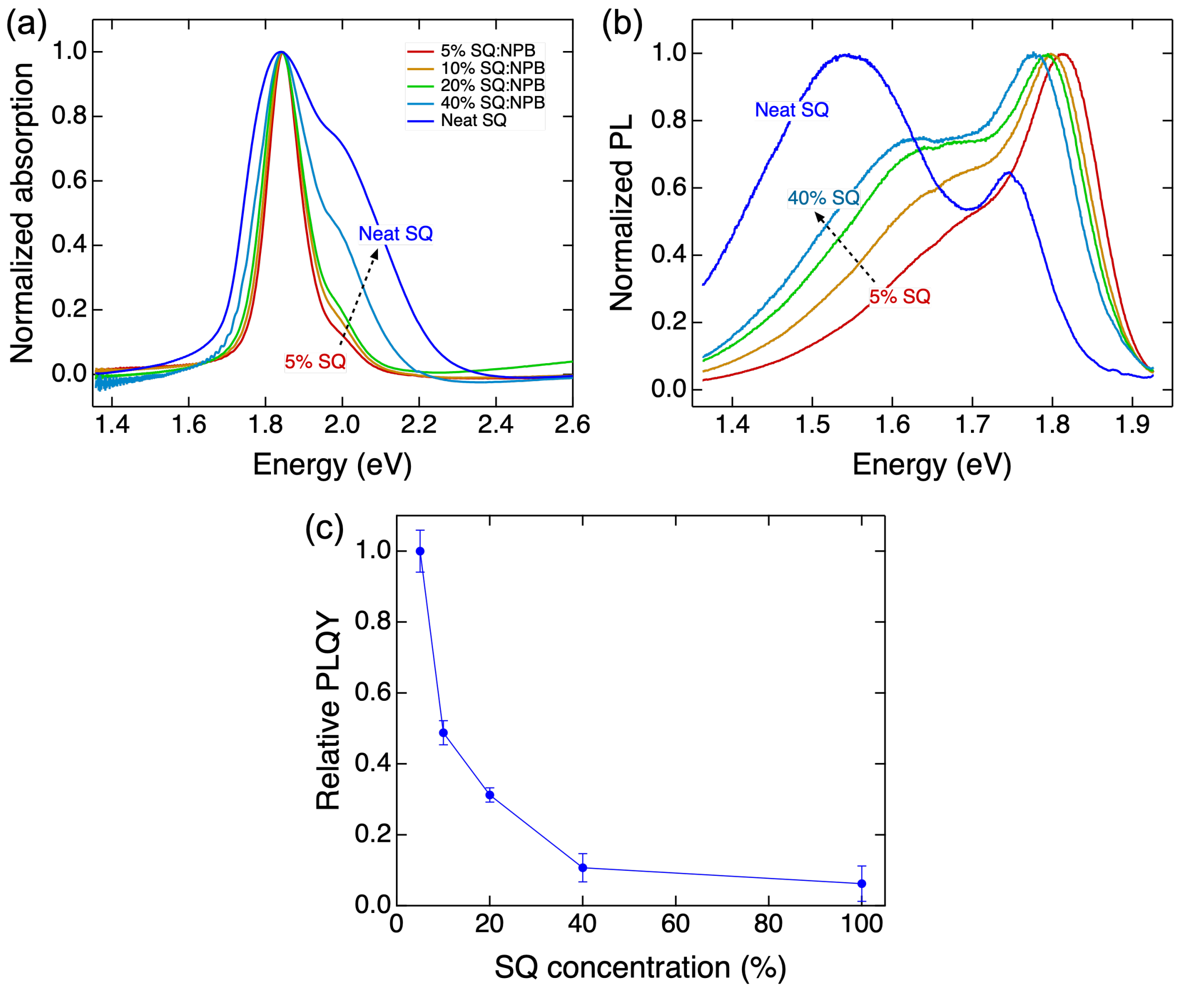

Figure S7. (a) Normalized absorbance spectra measured for 100 nm-thick SQ:NPB films with varying SQ concentration. A 100 nm-thick neat film of NPB (all on glass substrates) is placed in the reference arm of the spectrophotometer to minimize the impact of reflected light when calculating absorbance from transmission. **(b)** Normalized photoluminescence spectra of the same films using an excitation wavelength of 640 nm. **(c)** Relative photoluminescence quantum yield (PLQY) determined for these films accounting for the fraction of the excitation beam that is absorbed.

S8. Model including both H-aggregate states

The two-level model in the main text (Eqn. (1)) is readily expanded to include another (the upper H-aggregate) state at $E_3 = 1.94$ eV:

$$H_{\mathrm{SC}}(k) = \begin{bmatrix} E_1 & \mu_{12}F & \mu_{13}F & V_1 \\ \mu_{12}F & E_2 & \mu_{23}F & V_2 \\ \mu_{13}F & \mu_{23}F & E_3 & V_3 \\ V_1 & V_2 & V_3 & E_{\mathrm{ph,k}} \end{bmatrix}, \tag{S5}$$

which introduces additional cavity coupling (V_3) and excited state transition dipole parameters (μ_{13} and μ_{23}) to the problem. Figure S8 shows that this three-level model enables the magnitude of the UP shift to be increased without affecting the LP shift, which leads to better agreement with the data (compare the two- and three-level model results in Fig. S8c,d and Fig. S8e,f, respectively). Note that the broadening of each transition can also be included in Eqn. (S5) by using complex energies (where the imaginary part of $E_{1,2,3}$ and $E_{\mathrm{ph,k}}$ reflects their respective linewidth), though this has little effect on the results shown below and thus is neglected for simplicity. All of the parameters used in the two- and three-level models are summarized in Table S2 below.

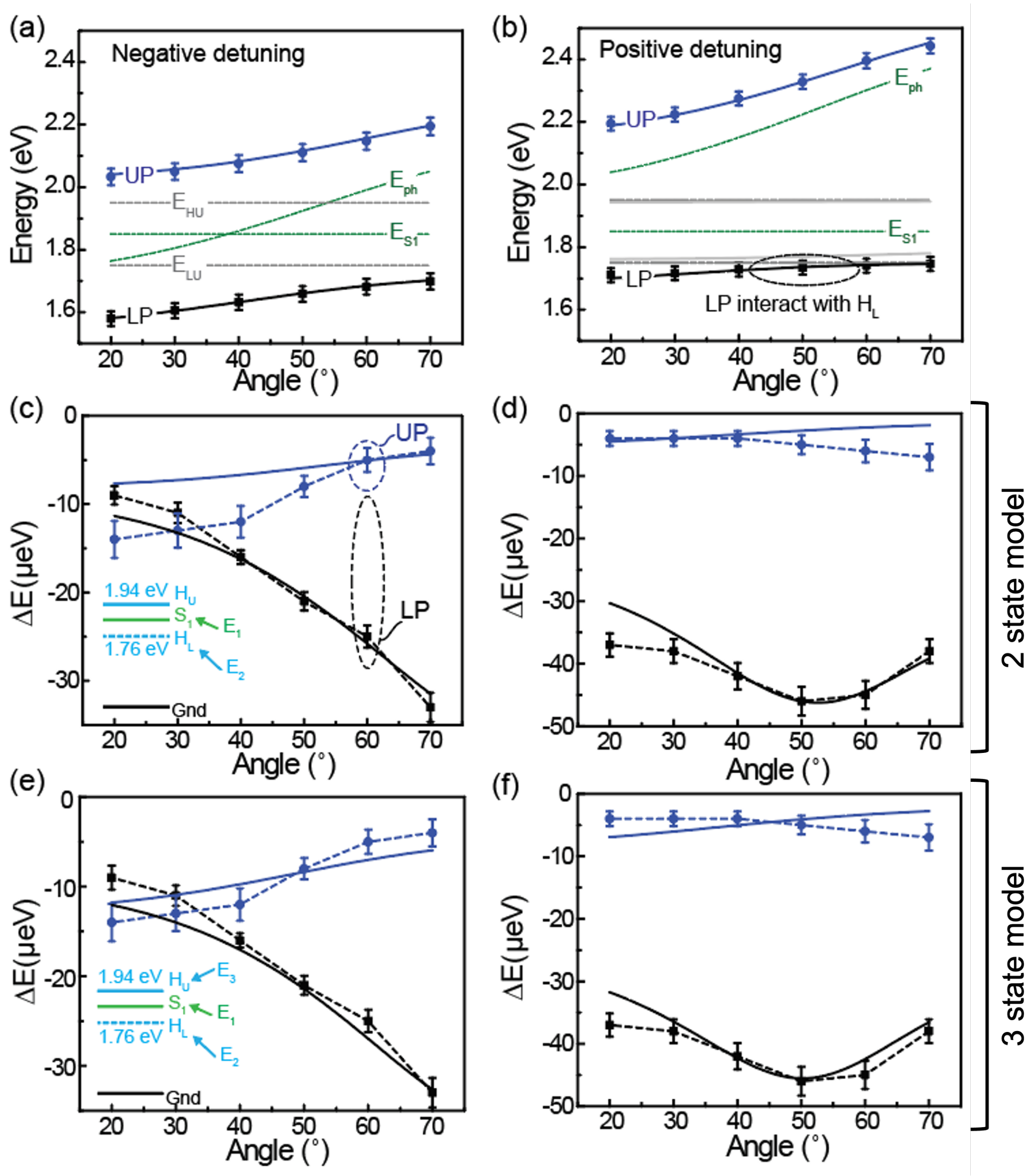

Figure S8. (a,b) Polariton dispersions of the negatively- and positively-detuned SQ:NPB cavities reproduced from Fig. 5a,b together with those calculated (solid lines) by diagonalizing Eqn. (S5). **(c,d)** Measured and predicted polariton energy shifts based on the two-level Hamiltonian (Eqn. (1)); the plots are reproduced from Fig. 5e,f. **(e,f)** Measured and predicted polariton energy shifts based on the three-level Hamiltonian (Eqn. (S5)).

Table S2. Hamiltonian model parameters for 40 wt% SQ:NPB cavities

	**2-level model**		**3-level model**	
**Cavity**	Neg. det.	Pos. det.	Neg. det.	Pos. det.
$\boldsymbol{E_{\mathrm{ph0}}}$ **(eV)**	1.73	2.01	1.73	2.01
$\boldsymbol{n_{\mathrm{cav}}}$	1.75		1.75	
$\boldsymbol{E_1}$ **(eV)**	1.85		1.85	
$\boldsymbol{E_2}$ **(eV)**	1.76		1.76	
$\boldsymbol{E_3}$ **(eV)**	--		1.94	
$\boldsymbol{V_1}$ **(eV)**	0.21		0.21	
$\boldsymbol{V_2}$ **(eV)**	0.04		0.04	
$\boldsymbol{V_3}$ **(eV)**	--		0.06	
$\boldsymbol{\mu_{12}}$ **(D)**	0.25		0.24	
$\boldsymbol{\mu_{13}}$ **(D)**	--		0.04	
$\boldsymbol{\mu_{23}}$ **(D)**	--		0.08	
$\boldsymbol{F}$ **(V cm^{-1})**	10^5		10^5	

One point of note regarding the transition dipole moments in Eqn. (1) and Eqn. (S5) is that they do not couple the monomolecular exciton transition to the H-aggregate states since the monomolecular exciton does not coexist on the same molecules that form an H-aggregate. Rather, the transition dipole moments refer to H-aggregates that possess bright states at approximately the monomolecular exciton energy[2] that can mix with the lower dark states via the applied field (these bright states exist for $N = 4s + 1$, where N is the number of monomers in the aggregate and $s > 0$ is an integer). In mixed films such as ours, there is likely a distribution of SQ molecule pairings that ranges from isolated molecules to H-aggregate dimers, trimers, and higher (this is evident in the absorption spectra of Fig. S7a, where both the monomolecular

exciton and upper H-aggregate transitions coexist at high SQ concentration) and thus the transition dipole moments in Eqn. (1) and Eqn. (S5) should be interpreted as effective values for the distribution.

S9. Effect of lower H-aggregate energy and oscillator strength

The bump in the LP shift at ~50º in Fig. S8d,f is associated with the point at which the LP mode crosses the lower H-aggregate state. Figure S9a highlights this point by varying the energy of the lower H-aggregate in Eqn. (1), which systematically changes the crossing point as illustrated in the inset, and in turn shifts the angle at which the bump occurs. Note that, since the H-aggregate has a weak, but finite coupling to the cavity mode (i.e. $V_2 \neq 0$), diagonalizing Eqn. (1) nominally predicts a weakly dispersive, mostly H-aggregate-like state that undergoes a mirror symmetric EA shift with respect to the LP in Fig. S9a (gray lines). This weakly coupled state is presumably not observed in reflectivity or EA because it is dominated by the much more intense LP feature. The coupling strength of the H-aggregate is, however, predicted to manifest in the linewidth of the LP EA bump, where a darker H-aggregate state produces a sharper bump as shown in Fig. S9b. While Fig. S9 motivates the importance of polariton-dark state crossing points for EA and likely other optical nonlinearities, we emphasize that a full understanding will require a rigorous theory for polariton EA that includes vibronic effects and can be used to fit reflectivity, transmission, and electroreflectance spectra directly from the system Hamiltonian; we are currently working toward this goal[4,5].

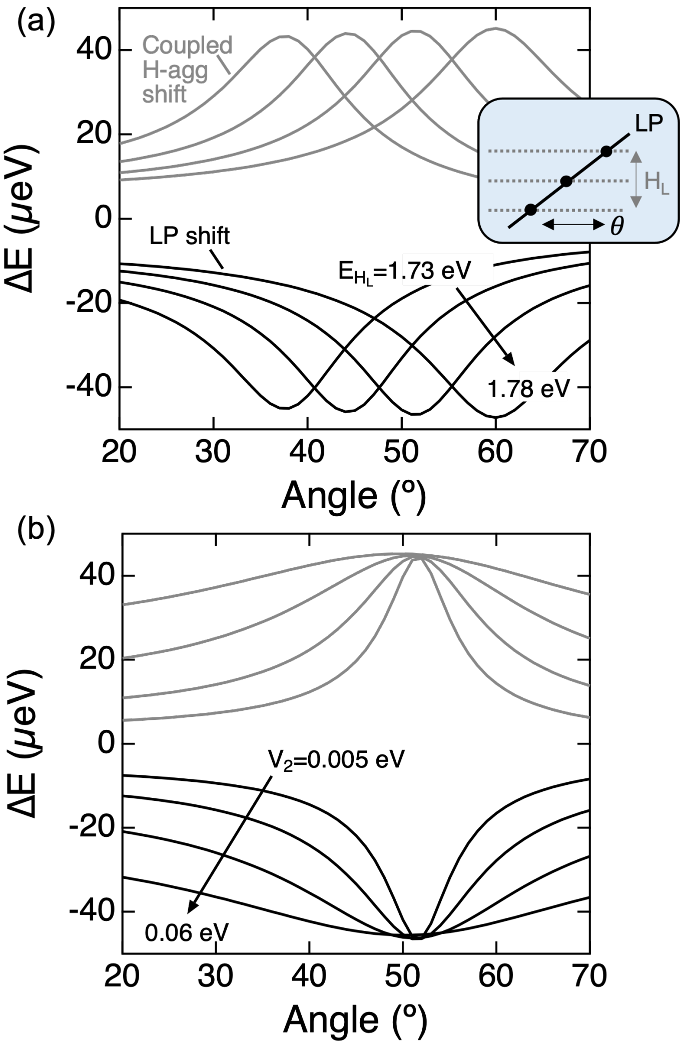

Figure S9. (a) Field-induced energy shift of the LP and coupled H-aggregate state predicted by Eqn. (1) for varying energies of the bare H-aggregate (E_2) in the vicinity of the nominal value of $E_2 = 1.76$ used in Fig. 5e,f. The inset illustrates how varying the bare H-aggregate energy changes the angle at which the LP mode crosses it. **(b)** Field-induced energy shift of these two states for varying H-aggregate-cavity coupling strengths (V_2).

S10. Impact of strong coupling on the field dependence of EA: SubPc cavities

Another interesting feature of Eqn. (1) is that it predicts a change in the field dependence of the EA signal when both transitions are strongly coupled to the cavity mode. In the absence of a cavity, Eqn. (1) reduces to:

$$H_0 = \begin{bmatrix} E_1 & \mu_{12}F \\ \mu_{12}F & E_2 \end{bmatrix}, \tag{S6}$$

and has eigenvalues of:

$$\lambda_+ \approx E_2 + \frac{(\mu_{12}F)^2}{E_2 - E_1}, \tag{S7a}$$

$$\lambda_- \approx E_1 - \frac{(\mu_{12}F)^2}{E_2 - E_1}, \tag{S7b}$$

assuming that the field perturbation is much smaller than the zero-field energy difference between the two states (i.e. $\mu_{12}F \ll E_2 - E_1$, assuming $E_2 > E_1$ for concreteness). The field-induced energy shift of each state is thus $\pm(\mu_{12}F)^2/(E_2 - E_1)$, which scales quadratically with the field strength as expected from standard perturbation theory. A different approach to obtain the same result follows from expressing the eigenvalues as the sum of a zero-field component (λ_{ZF}, which in this case is just E_1 and E_2) and the field-induced perturbation (δ_{F}) via $\lambda \rightarrow \lambda_{\mathrm{ZF}} + \delta_{\mathrm{F}}$. Writing the characteristic eigenvalue equation and then setting the terms with lowest order (i.e. linear) in δ_{F} equal to those involving the field, we obtain:

$$\delta_{\mathrm{F}} \approx \frac{(\mu_{\mathrm{ee'}}F)^2}{2\lambda_{\mathrm{ZF}} - E_1 - E_2}, \tag{S8}$$

which is equivalent to Eqn. (S7) above since λ_{ZF} is just E_1 or E_2.

When both transitions are strongly coupled to the same cavity mode per Eqn. (1) from the main text, the same perturbative approach can be used to obtain the field-induced shift of the polariton modes:

$$\delta_{\mathrm{F,SC}} = \frac{2V_1V_2(\mu_{12}F) - E_{\mathrm{ph,k}}(\mu_{12}F)^2}{3\lambda_{\mathrm{ZF}}^2 - 2\lambda_{\mathrm{ZF}}\left(E_{\mathrm{ph,k}} + E_1 + E_2\right) + E_1E_2 + E_1E_{\mathrm{ph,k}} + E_2E_{\mathrm{ph,k}} - V_1^2 - V_2^2}, \qquad \text{(S9)}$$

where now λ_{ZF} are the energies of the zero-field lower, middle, and upper polaritons. It is evident from Eqn. (S9) that when only one of the two transitions strongly couples to the cavity mode, the polariton energy shifts scale quadratically with field, but when both of them couple, the shifts become linear in the field since $V_1, V_2 \gg |\mu_{12}F|$ (e.g. $V_1, V_2 \sim 100$ meV vs. $\mu_{12}F \sim 100$ μeV). Physically, this nonzero $O(F)$ can be understood by applying the static field perturbation to the zeroth-order polariton states; since the latter carry amplitudes from excitons 1 and 2, the static field mixes them already at first-order perturbation theory.

To explore this prediction, we study the EA response of cavities containing SubPc diluted at 25 wt% into the wide-gap host material CBP. SubPc is unique in this context because it has a doubly-degenerate S_1 exciton transition[6] that should, in principle, fulfill the requirements for linear field scaling in Eqn. (S9). Its non-planar conformation also discourages aggregation, thereby minimizing the possibility of unforeseen aggregate state contributions to EA as in the case of SQ.

The impact of an applied field on the degenerate SubPc exciton transition is examined using the same TDDFT methodology as for SQ above. In vacuum, SubPc has C_3 symmetry and degenerate lowest energy S_1 and S_1' transitions. The dominant molecular orbital contributions to each transition are shown in Fig. S10. An applied electric field breaks the degeneracy of these

excited states, with a field of 0.01 atomic units (a.u., equivalent to ~51 MV cm^{-1}) leading to state splitting of 0.03-0.2 eV, depending on the direction of the field (see Table S3).

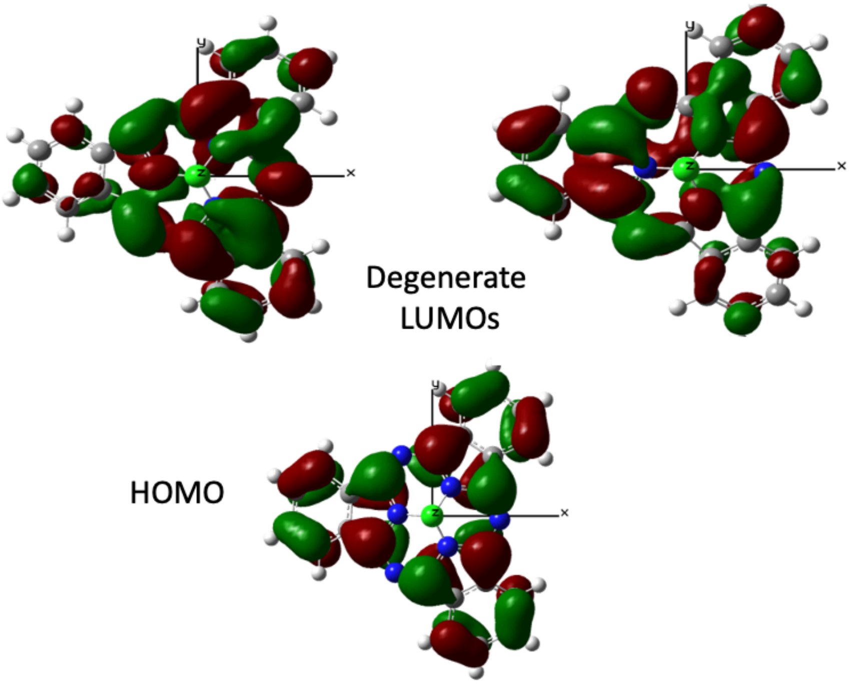

Figure S10. B3LYP/6-311+G*(2d,p) molecular orbitals for SubPc in vacuum. The S_1 transition is primarily HOMO to LUMO (left) and the S_1' transition is primarily HOMO to LUMO (right).

The transition dipole moment strength between S_1 and S_1', computed with the configuration interaction singles CIS/6-311+G(2d,p) method, is μ_x = 0.60 a.u. = 1.52 Debye, μ_y = 0.25 a.u. = 0.65 Debye, and μ_z = 0, indicating that a field applied along the *x* or *y* directions will couple the degenerate states, causing them to mix, with one state shifting up in energy and the other shifting down in energy.

Table S3. Field-off and field-on TD-B3LYP/6-311+G(2d,p) S_1 and S_1' excitation energies (eV), transition dipole moments (atomic units), and oscillator strengths for SubPc in vacuum

	**Field (a.u.)**	**ΔE (eV)**	**μ_x (a.u)**	**μ_y (a.u)**	**μ_z (a.u)**	**f**
**S_1**	0.0	2.47	-2.2557	0.0001	0.000	0.3074
**S_1'**	0.0	2.47	0.0001	2.2557	0.000	0.3074
**S_1**	X=+0.01	2.28	-1.3752	-1.8259	0.0097	0.2923
**S_1'**	X=+0.01	2.44	-1.6610	0.7098	-0.0132	0.1950
**S_1**	X=-0.01	2.28	-1.3753	1.8258	0.0097	0.2923
**S_1'**	X=-0.01	2.44	1.6609	0.7099	-0.0132	0.1949
**S_1**	Y=+0.01	2.24	2.2197	0.0001	0.0000	0.2699
**S_1'**	Y=+0.01	2.47	0.0001	-1.7572	-0.0111	0.1868
**S_1**	Y=-0.01	2.35	-0.0002	2.5123	0.0076	0.3633
**S_1'**	Y=-0.01	2.38	-1.5667	-0.0002	0.0000	0.1434
**S_1**	Z=+0.01	2.43	-2.2182	0.0001	0.0000	0.2931
**S_1'**	Z=+0.01	2.43	-0.0001	-2.2182	0.0000	0.2931
**S_1**	Z=-0.01	2.48	2.2797	0.0406	0.0000	0.3163
**S_1'**	Z=-0.01	2.48	0.0405	-2.2798	0.0000	0.3163

Both the ground and excited state dipole moments are primarily along the *z*-direction (the direction of the C_3 axis), with the excited state dipole moment increasing in the *y* and *z*-directions, leading to preferential stabilization of the excited state over the ground state if the field is aligned with the dipole moment.

The experimental data in Fig. S11 support these predictions. Figure S11a shows the extinction coefficient spectrum measured for a 25 wt% SubPc:CBP film, which exhibits a primary 0-0 exciton transition at ~2.12 eV and a vibronic shoulder at ~2.3 eV that technically involves several vibrations[6], but which we refer to as 0-1 for simplicity. As with the SQ devices in the main text, we initially characterize the angle-dependent reflectivity and electroreflectance of a half-cavity control device (120 nm ITO/5 nm MoO_3/130 nm 25 wt% SubPc:CBP/20 nm Ag) shown in Fig. S11b and S11c, respectively. Consistent with the TDDFT results above, we find good agreement with the transfer-matrix-simulated EA in Fig. S11c when we assume that the SubPc absorption derives from two, nearly degenerate electronic transitions, S_1 and S_1', that are each modeled by a pair of Lorentz oscillators (denoted by the purple and green dashed lines in Fig. S11a) that represent their respective 0-0 and 0-1 vibronics. The simulated electroreflectance spectra in the lower panel of Fig. S11c are produced by red-shifting and strengthening the S_1 pair of oscillators ($\Delta E = -85$ μeV, fractional amplitude increase $\Delta f/f = 10^{-4}$), while blue-shifting the S_1' pair of oscillators ($\Delta E = +42$ μeV). Although a change in broadening might also be expected given the change in static dipole moment predicted by the TDDFT calculations, it is not required to reproduce the data and thus must be a small effect compared to the energy and amplitude changes. We note as well that, although the excited state degeneracy is exact for an isolated SubPc molecule in Table S3 above, we have to assume a 30 meV zero-field splitting between the S_1 and S_1' oscillators in Fig. S11a in order to reproduce the EA data. This zero-field splitting presumably stems from molecular packing effects in the film that distort the SubPc nuclear framework and break the formal excited state degeneracy of the isolated molecule.

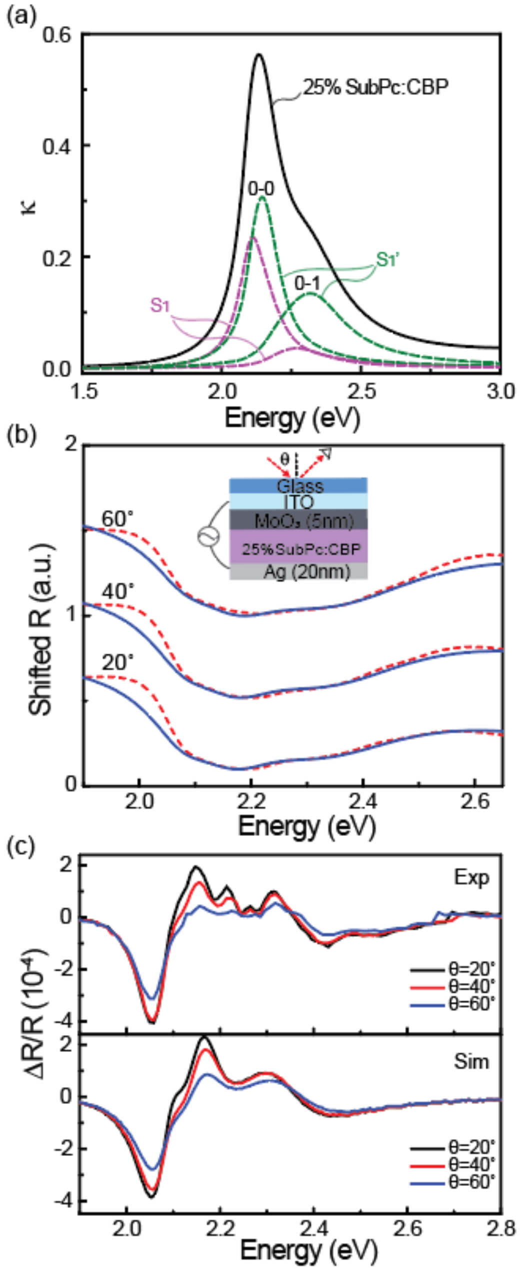

Figure S11. (a) Extinction coefficient dispersion measured for a 25 wt% SubPc:CBP film, together with the constituent oscillators used to represent the 0-0 and 0-1 vibronics of its near-degenerate S_1 (purple) and S_1' (green) exciton transitions. **(b)** Angle-dependent s-polarized reflectivity spectra measured for a half-cavity device (inset) together with corresponding transfer matrix simulations (dashed lines) based on the SubPc optical constants in (a). The spectra are

vertically offset for clarity. Panel **(c)** shows the measured (top) and simulated (bottom) EA spectra for this device that result from red-shifting and strengthening the S_1 pair of oscillators while blue-shifting the S_1' pair of oscillators.

Figure S12 presents a similar set of data for a negatively-detuned ($\Delta = -180$ meV with respect to the 0-0 SubPc transition) microcavity consisting of glass/ITO (120 nm)/Al (40 nm)/MoO_3 (5 nm)/25 wt% SubPc:CBP (130 nm)/Ag (20 nm). The angle-dependent reflectivity spectra in Fig. S12a display a clear anti-crossing between LP and UP modes, as well as two other non-dispersive reflectivity features marked by asterisks. The measured EA spectra in Fig. S12b are well described by the scaled reflectivity derivative (Fig. S12c) in the case of the LP feature, but not for the UP feature, presumably because the latter overlaps with one of the non-dispersive features in Fig. S12a. Nonetheless, if we focus on the well-resolved LP and maintain a simplified description of the system using Eqn. (1) with only the strongest (i.e. 0-0) S_1 and S_1' transitions, we find reasonable agreement with the data in Fig. S13 using the following parameters: $E_1 = 2.12$ eV, $E_2 = 2.15$ eV, $V_1 = 0.15$ eV, $V_2 = 0.21$ eV, $E_{\text{ph0}} = 1.94$ eV, $n_{\text{cav}} = 1.9$ eV, and $\mu_{12} = 0.05$ D, where E_1, E_2, and the ratio of the coupling strengths are fixed by the two 0-0 component oscillators in Fig. S11a (i.e. the ratio V_1/V_2 is equal to the square root of the ratio of the corresponding oscillator amplitudes).

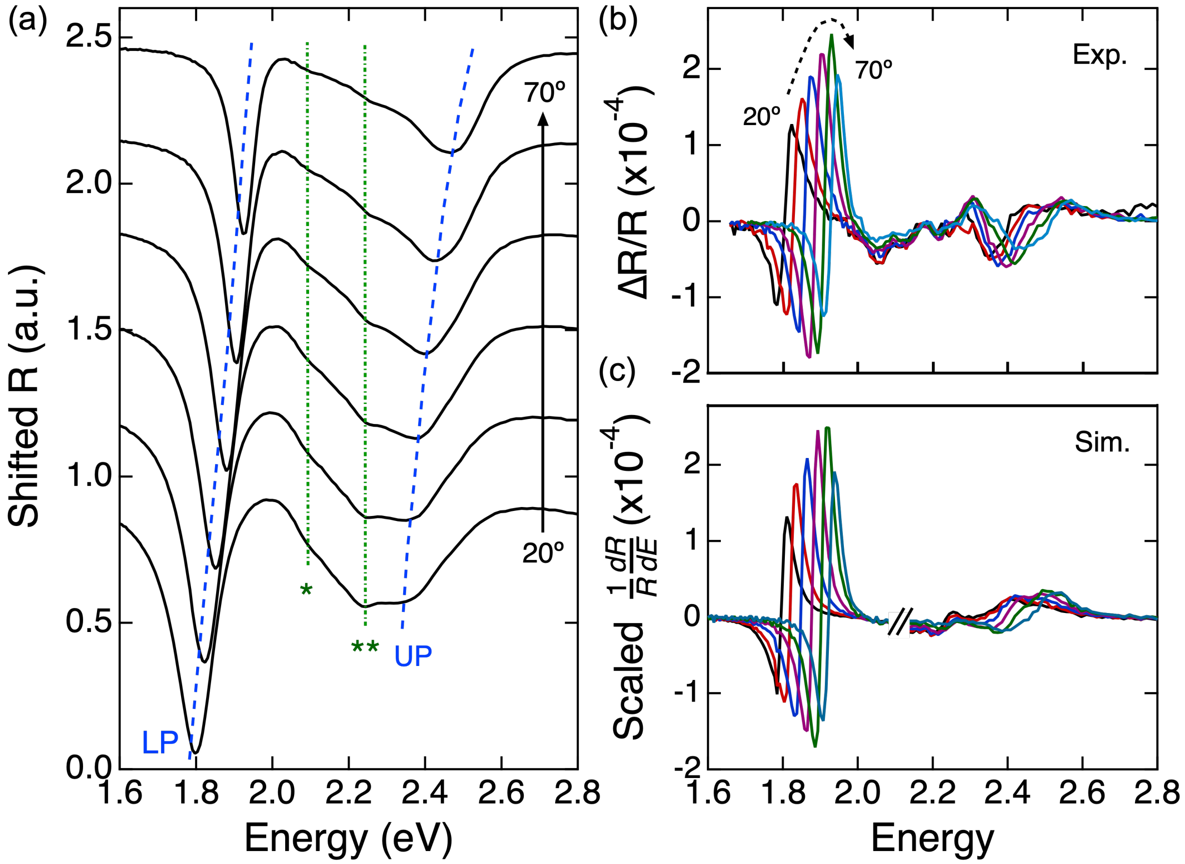

Figure S12. **(a)** Angle-dependent s-polarized reflectivity spectra measured for the negatively-detuned SubPc:CBP cavity device. The dispersive LP and UP modes are indicated by the blue dashed lines while the two non-dispersive shoulder features are marked by the dashed green lines. **(b)** Angle-dependent electroreflectance spectra measured for the same cavity. **(c)** Scaled derivatives of the experimental reflectivity data, $\frac{1}{R}\left(\frac{dR}{dE}\right)$, in (a). The LP and UP features are scaled independently (as indicated by the line break at 2.1 eV) to match the amplitude of the corresponding features in (b). The lineshape of the reflectivity derivative in the vicinity of the UP does not match the data owing to overlap between the UP and the non-dispersive feature in (a).

In addition to the LP and UP modes plotted in Fig. S13a, Eqn. (1) also predicts a non-dispersive middle polariton (MP) branch at approximately the energy of the bare transitions that roughly agrees with one of the non-dispersive features (*) identified in the reflectivity spectra from Fig. S12a.

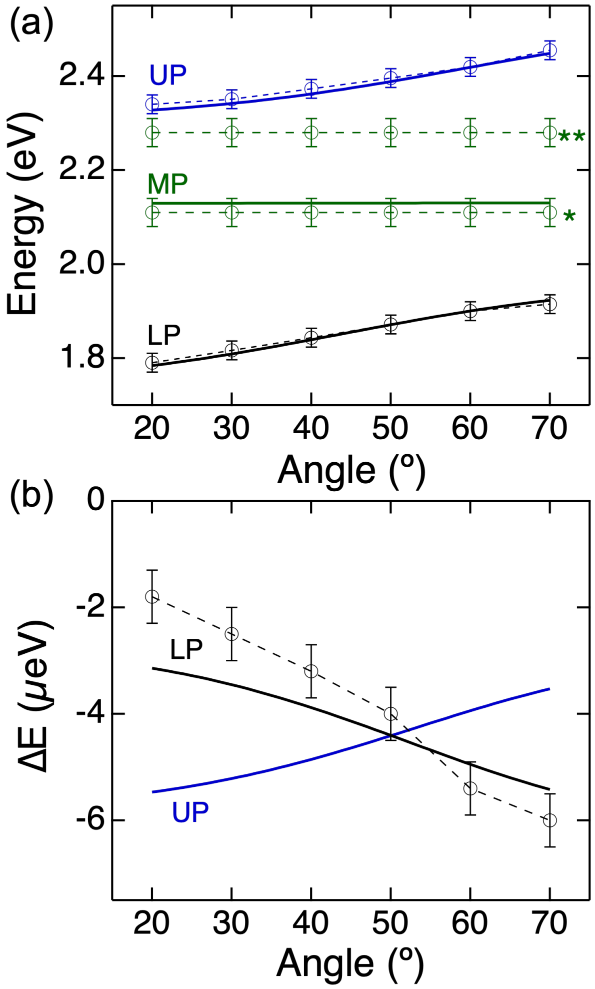

Figure S13. (a) Polariton dispersion relation for the negatively-detuned SubPc:CBP microcavity from Fig. S12. The LP and UP markers are determined from the experimental reflectivity minima and the solid lines are calculated using Eqn. (1) using the parameters given in the text. The green markers indicated by asterisks correspond to the reflectivity shoulder features identified in Fig. S12a. **(b)** Field-induced LP shift derived from the reflectivity derivative

analysis in Fig. S12c; the UP shift cannot be extracted because its reflectivity derivative lineshape does not match the experimental EA data. Solid lines show the LP and UP shifts predicted by Eqn. (1). The shift of the MP branch (not shown) is approximately independent of angle and equal to +8.6 μeV.

This is a general result of strong coupling degenerate transitions to a cavity mode and it therefore seems likely that the existence of the second non-dispersive feature in the data (**) is related to coupling of the degenerate 0-1 vibronics. Expanding Eqn. (1) to include the 0-1 transitions (a 5x5 matrix) supports this assertion; however, the larger number of parameters required to do so limits the transparency of the model and thus seems of limited value since it brings no new physical insight. Figure S13b shows that the field-induced LP shift is reasonably well captured by the model; comparison of the UP shift is unfortunately hindered by the difficulties with its reflectivity derivative lineshape noted above. For this reason, we focus our investigation on the field dependence of polariton EA exclusively on the LP mode.

To understand whether the field dependence of the LP EA differs from that of the half-cavity control, we monitor the ratio of their first-to-second harmonic EA amplitude as a function of DC bias. For a generalized EA response potentially having both linear and quadratic contributions to the field dependence, $\Delta R = AF + BF^2 + \cdots$, and assuming an applied electric field $F = F_{\mathrm{DC}} + F_{\mathrm{AC}}\sin(\omega t)$, it is straightforward to show that the ratio of the first-to-second harmonic magnitudes, $\Delta R_{1\omega}/\Delta R_{2\omega} = 2A/(BF_{\mathrm{AC}}) + 4F_{\mathrm{DC}}/F_{\mathrm{AC}}$. Thus, plotting $\Delta R_{1\omega}/\Delta R_{2\omega}$ versus F_{DC} for different fixed values of F_{AC} should produce a family of lines that all intercept the

x-axis at $F_{\mathrm{DC}} = -A/(2B)$, thereby providing a measure of the relative contributions of the linear and quadratic components of the EA response.

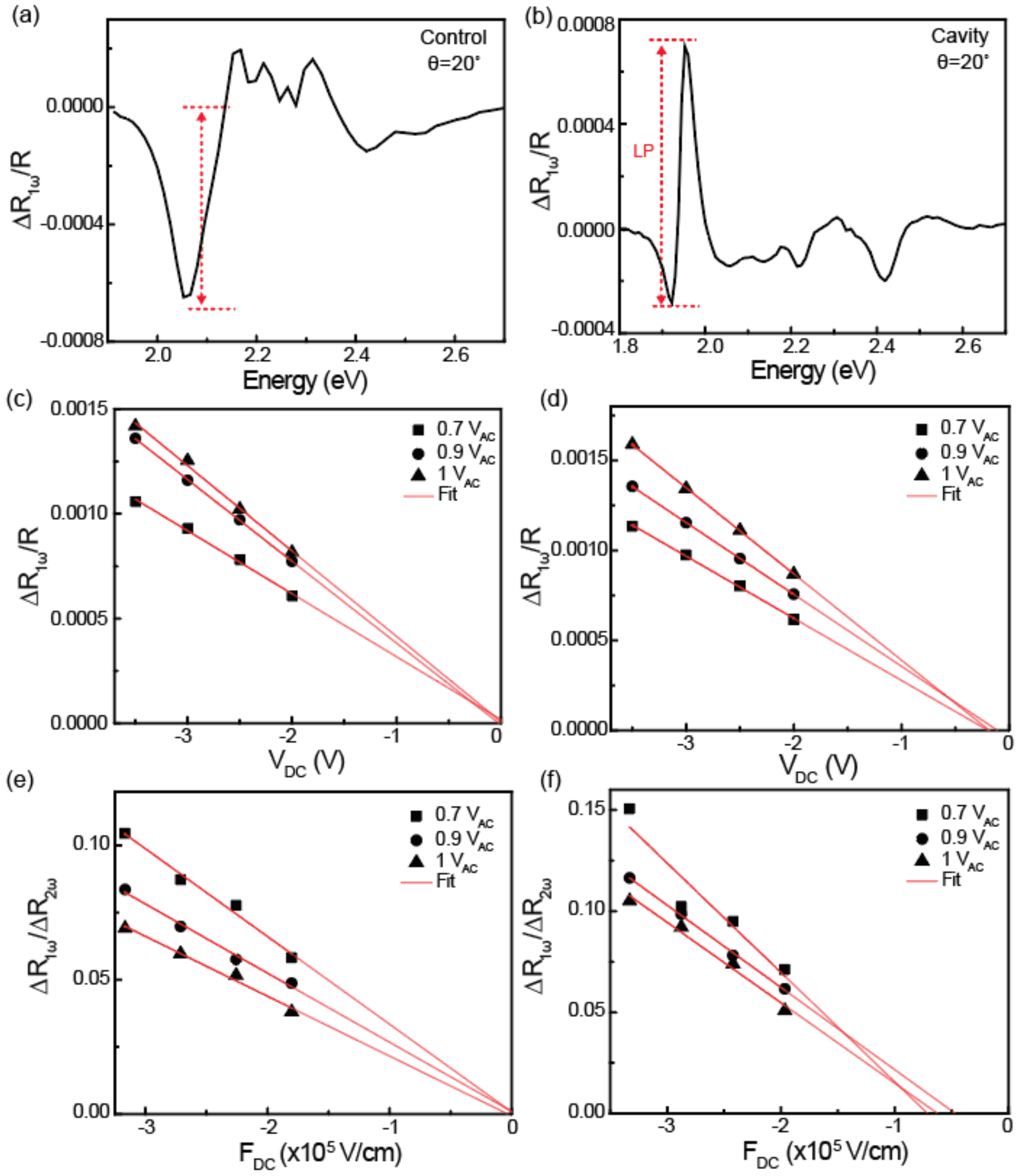

Figure S14. Electroreflectance spectra for a 25 wt% SubPc:CBP **(a)** half-cavity control device and **(b)** a positively-detuned cavity device ($\Delta = +125$ meV; the device structure is the same as in Fig. S11 except with a 110 nm-thick SubPc:CBP active layer) measured at 20º incidence angle

with s-polarization. Panels **(c)** and **(d)** respectively show how the red arrow amplitudes in (a) and (b) vary with DC bias for different AC modulation amplitudes. **(e)** Ratio of the first-to-second harmonic EA signal measured for the control device at different AC dither amplitudes; the red lines are linear regressions for each data set. **(f)** Analogous data for the cavity device.

Figure S14a and S14b show the (first harmonic) EA spectra of a half-cavity control sample and a positively-detuned ($\Delta = +125$ meV) cavity, respectively. Tracking the amplitude of the most prominent feature in each case (indicated by the red dashed lines) as a function of the DC bias and extrapolating the data to the x-axis in Fig. S14c,d subsequently yields the built-in potential, V_{bi}, for each device[7]. This in turn enables an accurate calculation of F_{DC} (i.e. $V_{\mathrm{DC}} + V_{\mathrm{bi}}$ divided by the organic layer thickness) for the plots of $\Delta R_{1\omega}/\Delta R_{2\omega}$ shown in Fig. S14e,f. While the results for the control device show a consistent intercept at the origin for all AC modulation amplitudes (implying that $A = 0$ and the field dependence is fully quadratic), the intercepts for the LP feature in the cavity device seem to cluster away from the origin, implying a non-zero linear contribution to the signal. There is, however, significant scatter in the LP intercepts and thus it is not possible for us to conclude with certainty whether the LP EA truly exhibits a different field dependence than the control, though it does seem clear that the LP field dependence has a significant quadratic contribution since otherwise there would be no second harmonic signal at all. A full understanding of the field dependence of polariton EA in relation to that predicted by Eqn. (S9) thus remains a key question for future work.

Supplementary References

1. Boyd, R. W. Nonlinear Optics. (Academic Press, 2020).
2. Hestand, N. J. & Spano, F. C. Expanded Theory of H- and J-Molecular Aggregates: The Effects of Vibronic Coupling and Intermolecular Charge Transfer. *Chem. Rev.* **118**, 7069–7163 (2018).
3. Zheng, C. *et al.* Contribution of Aggregate States and Energetic Disorder to a Squaraine System Targeted for Organic Photovoltaic Devices. *Langmuir* **31**, 7717–7726 (2015).
4. Qiu, L. *et al.* Molecular Polaritons Generated from Strong Coupling between CdSe Nanoplatelets and a Dielectric Optical Cavity. *J. Phys. Chem. Lett.* **12**, 5030–5038 (2021).
5. Spano, F. C. Optical microcavities enhance the exciton coherence length and eliminate vibronic coupling in J-aggregates. *J. Chem. Phys.* **142**, 184707 (2015).
6. Azarias, C., Pawelek, M. & Jacquemin, D. Structural and Optical Properties of Subporphyrinoids: A TD-DFT Study. *J. Phys. Chem. A* **121**, 4306–4317 (2017).
7. Campbell, I. H. *et al.* Measuring internal electric fields in organic light-emitting diodes using electroabsorption spectroscopy. *Polym. Adv. Technol.* **8**, 417–423 (1997).
